# Ion Channel Expression in Human Melanoma Samples: In Silico Identification and Experimental Validation of Molecular Targets

**DOI:** 10.3390/cancers11040446

**Published:** 2019-03-29

**Authors:** Daniela D’Arcangelo, Francesca Scatozza, Claudia Giampietri, Paolo Marchetti, Francesco Facchiano, Antonio Facchiano

**Affiliations:** 1 Istituto Dermopatico dell’Immacolata (IDI-IRCCS), 00167 Rome, Italy; d.darcangelo@idi.it (D.D.); f.scatozza@idi.it (F.S.); 2 Department of Anatomy, Histology, Forensic Medicine and Orthopedics, Unit of Histology and Medical Embryology, Sapienza University of Rome, 00161 Rome, Italy; claudia.giampietri@uniroma1.it; 3 Medical Oncology, Sapienza, University of Rome, 00161 Rome, Italy; paolo.marchetti@uniroma1.it; 4 Department of Oncology and Molecular Medicine, Istituto Superiore di Sanità (ISS), 00161 Rome, Italy; francesco.facchiano@iss.it

**Keywords:** *KCNN2*, ion channels, melanoma, miconazole

## Abstract

Expression of 328 ion channel genes was investigated, by in silico analysis, in 170 human melanoma samples and controls. Ninety-one members of this gene-family (i.e., about 28%) show a significant (*p* < 0.05) differential expression in melanoma- vs. nevi-biopsies, taken from the GEO database. ROC (receiver operating characteristic) analysis selected 20 genes as potential markers showing the highest discrimination ability of melanoma vs. nevi (AUC > 0.90 and *p* < 0.0001). These 20 genes underwent a first in silico-validation round in an independent patients-dataset from GEO. A second-in silico-validation step was then carried out on a third human dataset in Oncomine. Finally, five genes were validated, showing extremely high sensitivity and specificity in melanoma detection (>90% in most cases). Such five genes (namely, *SCNN1A*, *GJB3*, *KCNK7*, *GJB1*, *KCNN2*) are novel potential melanoma markers or molecular targets, never previously related to melanoma. The “druggable genome” analysis was then carried out. Miconazole, an antifungal drug commonly used in clinics, is known to target *KCNN2*, the best candidate among the five identified genes. Miconazole was then tested in vitro in proliferation assays; it dose-dependently inhibited proliferation up to 90% and potently induced cell-death in A-375 and SKMEL-28 melanoma cells, while it showed no effect in control cells. Moreover, specific silencing of *KCNN2* ion channel was achieved by siRNA transfection; under such condition miconazole strongly increases its anti-proliferative effect. In conclusion, the present study identified five ion channels that can potentially serve as sensitive and specific markers in human melanoma specimens and demonstrates that the antifungal drug miconazole, known to target one of the five identified ion channels, exerts strong and specific anti-melanoma effects in vitro.

## 1. Introduction

Ion channels play a key role in the physiology of any cell- and tissue-type; this makes ion channels ideal potential therapeutic targets [1]. In a way, they represent the tollbooths filtering the molecular flux on the highways connecting the intracellular to the extracellular environment. Given the exceptional role of ion channels in controlling almost any cellular function, drugs targeting ion channels may cover a market of several billion USD [2]. Thus, it is no surprise the large interest pharmaceutical companies give to ion channels as molecular targets in many different pathological conditions, such as pain relief [3], cardiovascular diseases [4,5,6], diabetes [7], infectious diseases such as Hepatitis C virus [8] and influenza virus [9], CNS diseases [10,11,12,13], and cystic fibrosis [14,15]. Increasing interest is currently given to ion channels in cancers since they have been indicated as potential drug targets in many cancer conditions [16,17,18]. We have recently reported an extensive ion channels expression analysis in several solid tumors demonstrating relevant and significant alterations in bladder cancer, glioblastoma, melanoma, breast cancer, and lung carcinoma, in more than 3000 patients [19], further indicating ion channels as potential therapeutic targets or molecular markers in cancer field. Indeed, ion channels expression has been related to clinical outcome in breast cancer [20] and the role of different ion channels has been recently demonstrated in lung cancer [21,22] as well as prostate cancer [23,24,25]. Mutation in glutamate receptors have been related to increased survival in malignant melanoma [26]. In addition, directly targeting mitochondrial potassium channels exerts a potent antitumor effect in vivo in melanoma and in pancreatic adenocarcinoma mouse models [27]. These data confirm the crucial role ion channels play in many cancer conditions including melanoma and prompted us to investigate the expression level of 328 ion channels in human melanoma samples looking for ion channels genes acting as potential markers and molecular targets in a melanoma set up.

## 2. Results

**Figure 1:**
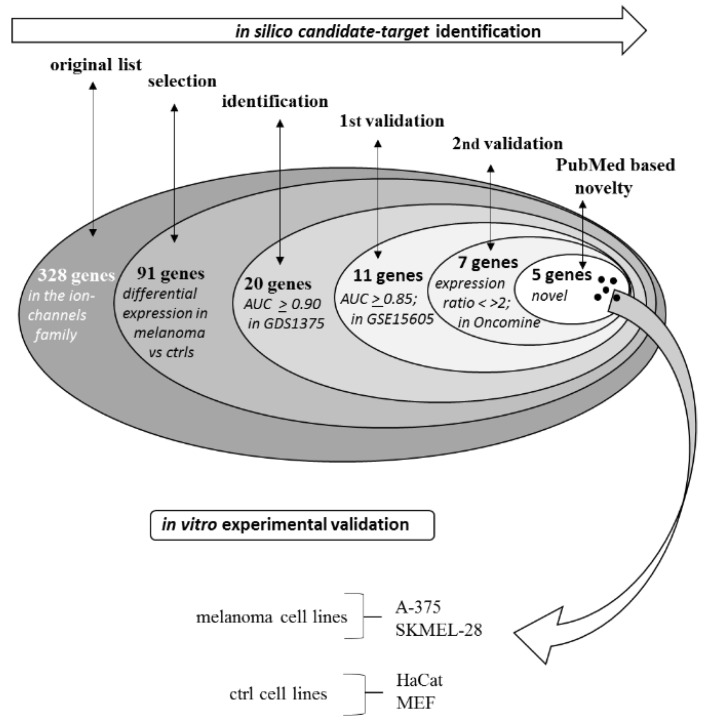
Procedure for the selection of the five best candidates and for the experimental validation.

The general procedure followed in the present study is summarized in Figure 1.

### 2.1. Selection Phase

The expression level of each of the 328 genes reported in Table 1 was compared in melanoma vs. controls. Table 2 reports the 91 genes showing a statistically different expression level in melanoma vs. controls (*p* < 0.05). The genes are reported in order of their *p* value, starting from the most significant (*p* > 10^−18^) down to the *p* > 0.05 threshold. Indeed, more than half of such genes fall within the *p* value below the 10^−4^ range, highlighting that a relevant number of members of the ion channels family are strongly and significantly modified in melanoma biopsies. For each gene, the computed fold change in melanoma vs. controls is also indicated, along with the AUC (area under the curve) according to the ROC (receiver operating characteristic) analysis. AUC indicates the ability to discriminate melanoma from nevi samples. Several genes show an extremely significant *p* value combined with a high fold change and high AUC value. Genes showing AUC > 0.90 and a *p* < 0.0001, i.e., a significant discriminating ability of at least 90%, were selected and are reported in Table 3, sorted according to the AUC level.

The analysis was carried out in GDS1375 dataset from the GEO database, containing data from 45 melanoma biopsies and 18 normal skin biopsies.

#### 2.1.1. First-Round Validation Step

The 20 genes having AUC ≥ 0.90 according to the expression levels reported in the GEO dataset GDS1375 were then analyzed in a different human samples dataset (GEO GSE15605) presenting data from 62 patients. Eleven genes showing an AUC level ≥ 0.85 in the second dataset were selected as genes validated in the 1st round validation, namely: *SCNN1A*, *ANO1*, *GJA1*, *GJB3*, *SCNN1B*, *GABRE*, *KCNK7*, *KCND3*, *KCNK1*, *GJB1*, *KCNN2* (see Table 3).

#### 2.1.2. Second-Round Validation Step

The 11 genes analyzed in GDS1375 and first-round validated in GSE15605 were then analyzed in a third dataset, namely the Riker dataset from Oncomine database. The seven genes showing an expression ratio in melanoma/controls <0.5 or >2 were then identified, namely: *SCNN1A*, *ANO1*, *GJB3*, *GABRE*, *KCNK7*, *GJB1*, *KCNN2* (see Table 3) and were considered second-round validated. Any known relation of these five genes with melanoma was then investigated according to a PubMed search, and they were all selected as novel genes:

*SCNN1A* (“*Sodium Channel Epithelial 1 Alpha Subunit*”, sodium channel, non-voltage-gated, amiloride sensitive),

*GJB3* (“*Gap Junction Protein Beta 3*”, gap-junction component, connexin gene family member),

*KCNK7* (“Potassium Two Pore Domain Channel Subfamily K Member 7”, potassium channel),

*GJB1* (“*Gap Junction Protein Beta 1*”, gap-junction component, connexin gene family member),

*KCNN2* (“Potassium Calcium-Activated Channel Subfamily N Member 2”, potassium channel).

**Figure 2:**
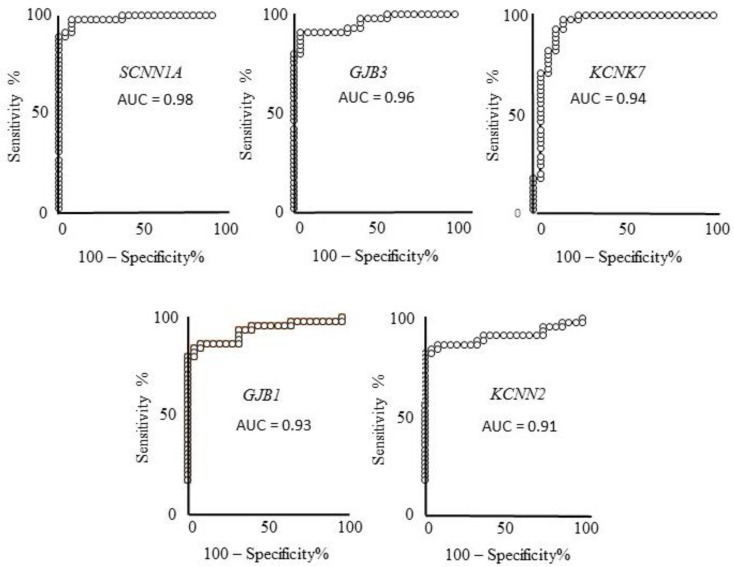
ROC analysis of the five genes expression showing a very high ability to discriminate controls from melanoma samples, validated in two samples datasets. These genes also show a strong expression ratio in the third dataset and are not reported to be related to melanoma according to PubMed searches. ROC analysis evaluates how the given measure (gene-expression level in this case) relates to sensitivity (ability to detect melanoma presence) and specificity (ability to detect melanoma absence). AUC (area under curve) of *SCNN1A*, *GJB3*, *KCNK7*, *GJB1*, and *KCNN2* indicate very high ability of their expression levels to discriminate melanoma from controls, namely 98%, 96%, 94%, 93%, and 91%, respectively.

No co-occurrence of their gene name or synonymous names is reported with “melanoma” word, in any field in PubMed searches (see Table 3, right end-side column). These five genes were then selected as the best novel candidates as melanoma markers and melanoma molecular targets. Figure 2 depicts the corresponding ROC curves of the five best candidates computed on the expression values reported in the GDS 1375 dataset. In all cases an AUC > 0.90 and *p* < 0.0001 is reported, i.e., a very high and significant ability to discriminate melanoma from healthy controls samples. The sensitivity and specificity values were computed as reported in the Methods section, indicating: 92% specificity and 97.7% sensitivity for *SCNN1A*; 96% specificity and 91% sensitivity for *GJB3*; 84% specificity and 97.7 sensitivity for *KCNK7*; 96% specificity and 84.4% sensitivity for *GJB1*; 100% specificity and 82.2% sensitivity for *KCNN2*.

### 2.2. Experimental Validation

According to the in silico screening and validations steps carried out and described above, *SCNN1A*, *GJB3*, *KCNK7*, *GJB1*, *KCNN2* genes were selected as best candidate melanoma markers and potential molecular targets. The five identified genes are down-regulated in three cases (namely *SCNN1A*, *GJB3*, *KCNK7*) and up-regulated in two cases (namely *GJB1* and *KCNN2*). Interestingly, both down- and up-regulated molecules may represent suitable molecular targets, exploiting the available blockers or activators, respectively. The five genes were then analyzed as targets of known FDA-approved drugs. The analysis was carried out on http://www.dgidb.org/search_interactions. Table 4 reports the results of this analysis, indicating a number of drugs (namely triamterene, amiloride, flufenamic acid, carbenoxolone, miconazole, tubucurarine, and bendroflumethiazide) known to target the identified ion channels. Main known tissue targets and pharmacological actions are also reported; such drugs act on muscles, joints, kidney, CNS, and also act on systemic targets. We focused our attention onto miconazole, an antifungal compound commonly used to treat skin infections. Miconazole has known skin distribution and dermatological pharmacokinetics; we, therefore, hypothesized it may be suitable for other skin diseases.

**Figure 3:**
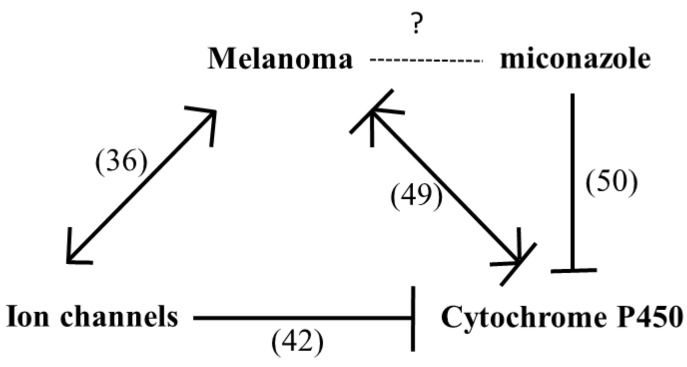
Chilibot analysis of Pubmed reported interactive relationships. According to the linguistic analysis carried out by Chilibot, miconazole is known to inhibit cytochrome P450; melanoma and cytochrome P450 are connected by both stimulatory and inhibitory relations; ion channels inhibit cytochrome P450 and stimulate melanoma. In parenthesis indicate number of abstracts found in PubMed is reported.

**Figure 4:**
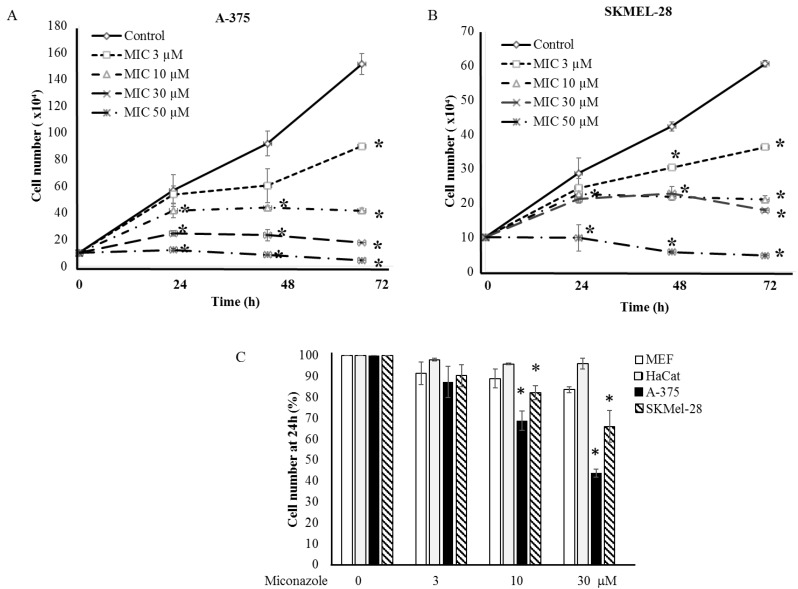
(**A**,**B**) Proliferation of A-375 (**A**) and SKMEL-28 (**B**) melanoma cells under miconazole treatment. * indicates *p* < 0.01; Miconazole strongly inhibits the growth of either melanoma cell lines in a dose-dependent manner; and (**C**) miconazole does not affect proliferation of control cells (HaCat keratinocytes and embryonic fibroblasts).

**Figure 5:**
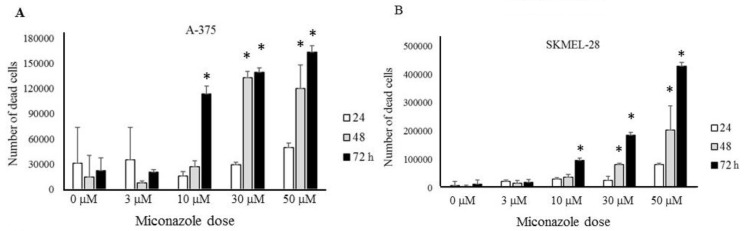
Quantification of cell death as function of miconazole dose and time of treatment, (**A**) A-375 (**B**) SKMEL-28. * indicates *p* < 0.01.

A Chilibot analysis identifies literature-reported functional relationships within user-defined terms. It identified the known functional relationships reported in literature among melanoma, ion channels, miconazole and cytochrome P450, one of the best characterized targets of miconazole. Chilibot analysis is reported in Figure 3, highlighting that miconazole inhibits cytochrome P450; melanoma and cytochrome P450 are connected by both stimulatory and inhibitory relations; potassium channels are known to inhibit cytochrome P450 [28] and their inhibitors are known to inhibit melanoma [29]. According to this scenario, a potassium channel inhibitor and cytochrome P450 inhibitor such as miconazole may significantly affect melanoma proliferation. Miconazole was then tested in vitro in a proliferation assay on two human melanoma cell lines, i.e., one more aggressive (namely A-375) and the other less aggressive (namely SKMEL-28). Proliferation was measured in the presence of 10% FCS. The results of a dose-dependent and time-dependent proliferation assay are reported in Figure 4A,B. Miconazole 10, 30, and 50 μM doses show a strong dose-dependent inhibition of serum-induced proliferation. Figure 4C shows that miconazole does not have any inhibitory effect on keratinocytes ad fibroblast control cells. Figure 5 reports the number of dead cells in the presence of 10% serum, indicating 30 and 50 μM as more potent cell-death inducers both in A-375 and in SKMEL-28 cells.

**Figure 6:**
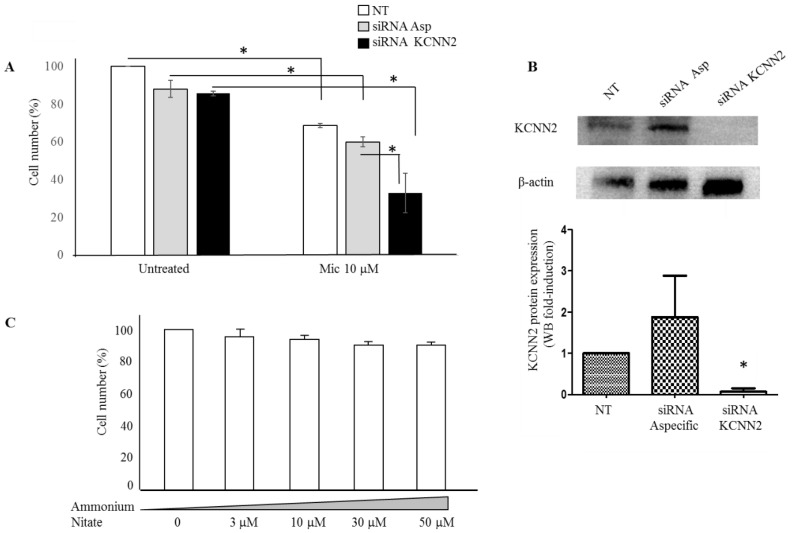
A-375 cells under *KCNN2*-siRNA and ammonium nitrate treatment. (**A**) Miconazole effect in A-375 cells transfected with siRNA-*KCNN2* and non-specific siRNA control. Bars without miconazole (left end of the graph) are expressed as % of untreated cells (NT); bars with miconazole (right end of the graph) are expressed as % of the corresponding control (NT with miconazole vs. NT without miconazole; siRNA Asp with miconazole vs. siRNA Asp without miconazole; siRNA *KCNN2* with miconazole vs. siRNA *KCNN2* without miconazole). Miconazole anti-proliferative effect is almost doubled in siRNA *KCNN2*-treated cells as compared to siRNA non-specific-treated cells. (**B**) KCNN2 protein expression in siRNA treated cells. siRNA *KCNN2* strongly reduces *KCNN2* expression in A-375 cells. (**C**) Ammonium nitrate has no anti-proliferative effect on A-375 cells; therefore, we may conclude that the anti-proliferative action of miconazole-nitrate is due to miconazole. * indicate *p* < 0.01.

We, therefore, investigated whether the anti-proliferation effect of miconazole may be affected by specifically modulating *KCNN2* expression. Figure 6 reports the strong increase of miconazole anti-proliferation effect upon *KCNN2* siRNA silencing (Figure 6A). Western blot analysis and densitometry quantification is reported in Figure 6B and confirms the strong downregulation of *KCNN2* achieved by siRNA treatment. Since the molecular form of miconazole used in the present study is miconazole-nitrate, as control the effect of ammonium-nitrate was also evaluated. Figure 6C shows that nitrate, at the same doses used for miconazole, does not show any significant effect.

### 2.3. Mechanisms Underlying Miconazole Action

Miconazole is reported to primarily target 14-α demethylase, a cytochrome P-450 enzyme involved in conversion of lanosterol to ergosterol, an essential component of the fungal cell membrane (see Drug Bank at https://www.drugbank.ca/drugs/DB01110). Nevertheless, it also inhibits several other targets, including endothelial nitric oxide synthase and inducible nitric oxide synthase as well as several potassium channels, namely: calcium-activated potassium channel subunit alpha-1, calcium-activated potassium channel subunit beta-1, calcium-activated potassium channel subunit beta-2, calcium-activated potassium channel subunit beta-3, calcium-activated potassium channel subunit beta-4, intermediate conductance calcium-activated potassium channel protein 4, small conductance calcium-activated potassium channel protein 1, small conductance calcium-activated potassium channel protein 2, small conductance calcium-activated potassium channel protein 3, potassium voltage-gated channel subfamily H member 2, potassium voltage-gated channel subfamily H member 6, and potassium voltage-gated channel subfamily H member 7. It is also a partial agonist of nuclear receptor subfamily 1 group I member 2. According to a STRING analysis carried out at www.string-db.org, such proteins are strongly involved in arginine metabolism, potassium transport, control of guanylate cyclase, nitric oxide synthesis, blood circulation and synaptic transmission. Table 5 highlights the top 10 biological processes enriched in a statistically significant way by analyzing the above reported proteins, known miconazole targets.

## 3. Discussion

Ion channels have been shown to play a role in melanoma biology [42]. The number of studies relating ion channels to melanoma has increased in the last few years and ion channels are now recognized as potential co-targets in the new melanoma therapeutic strategies currently under continuous development [43]. We have previously reported strong and significant expression changes of several ion channels in solid tumors including glioblastoma, breast cancer, lung cancer, bladder cancer, and melanoma [19] and also proposed a possible role of ion channels in brain metastases onset [44]. Figure 3 of the present study also underlines that several studies identify reciprocal stimulatory relationships of melanoma and ion channels. The present study presents for the first time an extensive analysis of ion channels expression in human melanoma biopsies indicating a number of potential highly effective markers accurately validated in silico and never previously related to melanoma. An in silico procedure (summarized in Figure 1), after an initial screening on a first human biopsies dataset, progressively leads to the selection of five genes in a double-validation step carried out in two more human melanoma-patients datasets. The five ion channels selected as best candidates and never previously directly related to melanoma (namely *SCNN1A*, *GJB3*, *KCNK7*, *GJB1*, *KCNN2*) show very high AUC values (>0.90 in all cases) and very high specificity and sensitivity values (>90% in most cases). In a few cases these genes have been related to other cancers such as lung cancer [45], breast cancer [46], thyroid cancer [47], and ovarian cancer [48]. On the other hand, mir125B, which is known to target the *SCNN1A* gene [49] has been reported to control melanoma progression [50].

We have previously published a study involving a pure in silico four-step validation procedure carried out on autophagy-related genes in more than 500 melanoma patients [51]. In that study we demonstrated that many autophagy-related genes undergo relevant and significant expression changes in melanoma biopsies as compared to controls, and three genes, namely *WIP1*, *PEX3* and *BAG1*, show impressive melanoma markers features, such as very high AUC values and sensibility and specificity values. Interestingly enough, ion channels recently emerged as key regulators of autophagy [52], further extending their potential clinical applications. We, therefore, underline here that ion channels may represent suitable molecular targets for novel therapeutic/diagnostic approaches in the melanoma field. The current study investigates this hypothesis. By analyzing ion channels expression in 170 human melanoma biopsies, we show that an accurate preliminary in silico validation is able to identify candidates as relevant markers or molecular targets (see Table 3). A number of drugs, commonly used in clinics in different pathological conditions, are known to target the 5 best candidates identified. This allowed us to hypothesize novel potential clinical applications in a melanoma set up, for the drugs indicated in Table 3, within a drug repositioning strategy. Although all drugs listed in Table 4 may be potentially effective, we focused our attention on miconazole, given its known skin-targeting properties [53]. Miconazole is a known cytochrome P-450 inhibitor targeting ion channels including KCNN2 [38,54]. In the clinical practice it is commonly used as an antifungal topic compound in skin infections or for systemic infections, and, within the drugs reported in Table 3, sounds as the best candidate for skin-related lesions or systemic lesions of skin origin such as melanoma. Further, miconazole targets the *KCNN2* gene; this gene appears to be the best candidate among the five identified in Table 3, being the only one with AUC > 0.90 in both screening phase and first validation phase, and with very high melanoma vs. ctrl expression ratios (see Table 3). Figure 4 of the present study demonstrates that miconazole strongly reduces (up to 90%) the serum-induced proliferation of A-375 (more-aggressive) and SKMEL-28 (less-aggressive) melanoma cells [55]. A375 and SK-MEL-28 cells are largely studied human melanoma cells for their different aggressiveness and malignancy. We have recently correlated their aggressive phenotype with different molecular profiles [56]. In both cell types high levels of p53 have been demonstrated [57], while A-375 express higher Bax level [58]. Figure 4 also shows that the anti-proliferation effect of miconazole appears to be specific for melanoma cells, since proliferation of ctrl cells (keratinocytes and fibroblasts) is not affected. All together such data suggest a possible topical application of miconazole in the skin treatment of the excised primary melanoma.

Figure 5 shows that miconazole also induces a strong cell-death, possibly suggesting adjuvant applications in metastatic stages. Miconazole has been previously shown to have some anti-proliferative effect in mouse melanoma cells (about 50% inhibitory action) and a mild anti-melanogenesis effect [59], while we show for the first time a much stronger activity both as anti-proliferation and as cell-death inducer in human cells lines, particularly in A-375 cells, known to have an aggressive phenotype. Antitumor effects of miconazole have been previously reported in cancers setup different than melanoma [60,61,62,63], while the present study represents the first evidence indicating miconazole strong anti-proliferative and death-inducing activity in melanoma cells. Interestingly, we demonstrate in the present study that the *KCNN2* ion channel strongly modulates miconazole anti-melanoma effect. In fact, in A375 cells, specific silencing by siRNA-*KCNN2* leads to a strong increase of miconazole anti-proliferative effect.

Interestingly, miconazole, while known as a relevant antifungal drug, is also known to recognizes eukaryotic targets including several potassium channels. It has shown to induce cytoprotective effect under hypoxia conditions, likely by inhibiting HMGB1 and IL-8 release in Caco-2 intestinal cells [64]. Further, it has been shown to induce post-ischemic neurogenesis in rats [65], immune response in fish [66], to have promising anti Alzheimer’s disease activity [67] and to interact with the anticoagulant drug warfarin [68]. Further investigation on the underlying mechanism are currently under investigation. In agreement with findings of the present study, other antifungal compounds, namely itraconazole, show potent anti-melanoma action in vivo and in vitro on A-375 and SKMEL-28 cells, down-regulating different pathways including PI3K/mTOR [69]. Noteworthy, increased melanogenesis is a frequent sing in fungal infections [70], fungal infections in some cases mimicking melanoma lesions [71]. Accordingly, previous studies report anti-melanogenesis action of antifungal compounds such as clotrimazole by interfering on ERK and PI3K/AKT activity [72]. Table 5 underlines that the known targets of miconazole are primarily involved in potassium transport, arginine catabolism as well as guanylate cyclase activity and nitric oxide synthesis.

In conclusion all together these evidences support the role of ion channels in the melanoma setup and future investigation on anti-melanoma effect in vivo of miconazole and other anti-fungal compounds.

## 4. Materials and Methods

Ion channels investigated in the current study were taken from the list according to HUGO Gene Nomenclature Committee (HGNC), European Bioinformatics Institute (EMBL-EBI) at https://www.genenames.org/data/genegroup/#!/group/177. The ion channels list includes 328 genes and is reported in Table 1 in alphabetic order. The general procedure followed in the present study is depicted in Figure 1. It consists of a preliminary in silico phase (screening phase, first-round and second-round validation steps, and novelty assessment) and of a following experimental validation of one of the identified molecular targets in proliferation/cell-death assays.

In silico steps: screening phase and two validation steps. The screening and the two validation steps were carried out onto a total of 170 patients (63 in the screening phase + 62 in the first round validation + 45 in the third round validation).

Selection step: in silico selection of suitable melanoma markers. Expression of the 328 ion channels genes reported in Table 1 was investigated in a collection of human melanoma biopsies and controls. Namely, the melanoma GDS1375 dataset [73] (Talantov et al, 2005) from the GEO database (https://www.ncbi.nlm.nih.gov/gds/) was chosen, containing expression data from 63 samples (45 melanoma-patients and 18 nevi-patients) with free data download. The significance of the differential expression was evaluated according to Student t test analysis and fold change. ROC analysis, the well accepted test for binary assessments [74], was then performed to measure how effective is the expression-level of any given gene to discriminate healthy- from melanoma-biopsies. The computed area under curve (AUC) value ranges from 0.5 to 1, indicating a minimum of 50% to a maximum of 100% discrimination ability. Other datasets in the GEO database are available, such as GDS1989 and GDS1965, but with only few samples. The GDS1375 dataset used in this selection step and the GSE15605 dataset used in the following first validation step, were chosen due to the large number of melanoma biopsies and control biopsies. Transcriptomic data are from Affymetrix Human Genome U133A Array and from Affymetrix Human Genome U133 Plus 2.0 Array platforms, respectively.

In silico first validation step: validation of identified genes was carried out on a different GEO dataset, namely GSE15605 [75] containing expression data form 62 patients-biopsies (namely 16 controls, 46 primary melanoma and 12 metastatic melanoma samples). The 20 genes showing AUC > 0.90 computed according to the GDS1375 dataset, were analyzed in the GSE15605 dataset and were considered validated if the computed AUC was >0.85. Under such condition, 11 genes were validated.

In silico second validation step: a further validation of the genes passing the screening and the first validation step was carried out in the Riker melanoma dataset [76] within the Oncomine database (www.oncomine.org). Such dataset contains 45 human samples (40 metastatic melanoma samples and five controls) and reports the expression values as log2-median centered intensity. This value was used to calculate the ratio of melanoma vs. normal skin groups. A ratio higher than 2 or lower than 0.5 was considered effective to validate. Seven genes passed the second validation step. Five of these seven genes (namely: *SCNN1A*, *GJB3*, *KCNK7*, *GJB1*, and *KCNN2*) show not-previously recognized relation with melanoma, according to a PubMed search carried out in September 2018, highlighting any co-occurrence of any gene-name and “melanoma” words in ALL fields. Therefore, these five genes were considered novel in silico validated melanoma markers and potential therapeutic targets.

### 4.1. In Vitro Experimental Validation: Analysis of Potential “Druggability”

Drugs potentially targeting the in silico validated genes *SCNN1A*, *GJB3*, *KCNK7*, *GJB1*, and *KCNN2* were then investigated on the database available at http://www.dgidb.org/search_interactions. The analysis allows to identify FDA-approved drugs known to target the given genes.

### 4.2. Chilibot Analysis

Known functional interactions were investigated by Chilibot analysis (www.chilibot.net) [77]. Chilibot identifies literature–reported relationships within user-defined terms. This is achieved by looking at their co-existence in the same sentence within Pubmed abstracts. Such procedure identifies closer relations as compared to a plain Pubmed search. Chilibot then associates same-sentence co-occurrence to stimulatory- or inhibitory- or non-interactive relationships. A pairwise search was carried out indicating “melanoma”, “ion channels”, “miconazole” and “cytochrome P450” terms. The “advanced options” button was turned on, to account for all known synonyms of the given terms and minimize false negative findings (i.e., using all known synonyms in the search reduces the risk of ignoring abstracts containing synonyms of the given term) and the analysis was performed on the maximum number of abstracts possible (i.e., 50 abstracts).

### 4.3. Cell Culture and Melanoma Cells In Vitro Proliferation Assay

Cells were used from the 3rd to 5th passages. SKMEL-28 and A-375 melanoma cells were from ATCC (Manassas, VA, USA) and were maintained in complete medium Dulbecco’s modified Eagle’s medium (DMEM; Hyclone, South Logan, UT, USA) and in complete medium Minimum Essential medium Eagle (MEM; Hyclone, South Logan, UT, USA) supplemented with 10% FBS (Hyclone), 2 mM L-glutamine and 100 IU/mL penicillin/streptomycin (Invitrogen, Carlsbad, CA, USA) respectively in humidified 5% CO_2_ atmosphere at 37 °C as described [78,79]. Human keratinocytes (HaCat) and mouse embryonic fibroblasts (MEF cells) were from American Type Culture Collection (ATCC).

In some experiments, A-375 cells were treated with 3, 10, 30, and 50 µM of ammonium nitrate (Merck, Darmstadt, Germany) as control of miconazole-nitrate, in 24 h proliferation assays.

The culture medium was changed every three days and when cells were sub-confluent; monolayers were harvested by 1 min exposure to 0.1% Trypsin-EDTA (Life Technologies Inc, Carlsbad, CA, USA).

Miconazole nitrate was from CliniSciences (Paris, France). SKMEL-28 and A-375 cells were plated at 10 × 10^5^ and 8 × 10^4^ respectively, in p35 plates dishes at time 0. Then cells were starved for 18 h in serum-free medium and the next day treated with 3, 10, 30, and 50 µM of miconazole in complete fresh medium containing 10% FCS and DMSO at a final concentration of 50 µM. Control cells were treated with complete medium containing 10% FCS and DMSO at a final concentration of 50 µM. The effect of miconazole nitrate on cells proliferation in vitro was measured by directly counting the number of cells. At the end of incubation time (24, 48, and 72 h), cells were harvested and number of live cells and dead cells was counted. Dead cells were quantified by trypan blue incorporation.

### 4.4. KCNN2 Silencing by siRNA

One day after plating (4 × 10^4^ cells/mL), A-375 cells were transfected with Lipofectamine 2000 (Invitrogen) plus 50 nM small interfering RNA (siRNA) targeting *KCNN2* gene (siRNA *KCNN2*) (Santa Cruz, CA, USA) or with the corresponding non-specific control siRNA (siRNA non-specific) (ThermoFisher, Waltham, MA, USA). After 5-h transfection, supernatant was discarded and fresh complete medium was added. The next day, *KCNN2* expression in transfected cells was measured by Western blot analysis and miconazole treatment (10 μM) was started. After 24 h of miconazole treatment, cell proliferation was measured.

### 4.5. Western Blot Analysis

Cells were washed with cold PBS and then cell lysates were prepared in cell lysis buffer (Cell Signaling, Denvers, MA, USA), 1% sodium dodecylsulphate-polyacrylamide (SDS) (Sigma-Aldrich, St Louis, MO, USA) and 1 mM phenylmethanesulfonyl fluoride solution (PMSF) (Sigma-Aldrich, St Louis, MO, USA). Protein concentration was measured by a bicinchoninic acid protein assay kit (Pierce, Rockford, IL, USA) according to manufacturer’s instructions and then equal amounts of proteins (25 µg) were subjected to sodium dodecylsulphate-polyacrylamide gel electrophoresis (SDS-PAGE) and transferred onto nitrocellulose membrane (GE Healthcare, Life science, Wien, Austria). Membranes were saturated with 5% non-fat dry milk in T-TBS, incubated with the primary antibody overnight, and subsequently with horseradish peroxidase-conjugated (HRP) secondary antibody for 1 h at room temperature. Membranes were washed with T-TBS and developed using the chemiluminescence system (ECL Advance, Amersham Biosciences, San Diego, CA, USA). Antibody used: anti KCNN2/SK2 antibody was from Abcam (San Francisco, CA, USA); anti-β-actin was from Sigma (St Louis, MO, USA). Secondary antibody was HRP-goat anti-rabbit from Bio-Rad (Hercules, CA, USA). The intensity of Western blot bands was quantified by Bio-RAD Chemidoc Gel Imaging System with Image Lab 5.2.1. software (Bio-RAD, Hercules, CA, USA).

### 4.6. STRING Analysis

STRING analysis at https://string-db.org/ was performed to identify the biological processes most likely affected by the genes known to be miconazole targets. The “multiple proteins” search was carried out.

### 4.7. Statistics

Statistical significance was computed according to the Student’s *t*-test; *p* < 0.05 was chosen as significance threshold. ROC analysis was carried out with GraphPad Prism version 5 (GraphPad Software, San Diego, CA, USA, www.graphpad.com).

The sensitivity and specificity values were computed as previously reported [51] as the values corresponding to highest sensitivity-specificity product in each ROC curve data.

## 5. Conclusions

In conclusion, in the present study we show that many ion channels genes taken from the ion channels families (i.e., 91 out of 328 investigated genes) are differently expressed in melanoma vs. control human biopsies, according to an in silico analysis on 170 human samples. Five such genes (namely: *SCNN1A*, *GJB3*, *KCNK7*, *GJB1*, *KCNN2*) were identified as potential strong melanoma markers that had never been identified before. By in vitro experiments, one gene (namely: *KCNN2*) has been validated as a relevant molecular target in melanoma cell lines. Indeed, the antifungal drug miconazole shows an extremely high anti-proliferation activity in melanoma cell lines, mediated by *KCNN2*. We, therefore, conclude that ion channels are strongly involved in melanoma and that miconazole may exert a potent anti-melanoma activity.

## Figures and Tables

**Table 1:** Ion channels genes investigated in the present study, selected according to HUGO Gene Nomenclature Committee at https://www.genenames.org/cgi-bin/genefamilies/.

No.	Gene Name	No.	Gene Name	No.	Gene Name	No.	Gene Name	No.	Gene Name	No.	Gene Name
1	*ANO1*	56	*CACNG5*	111	*GABRB1*	166	*GRIN2D*	221	*KCNJ2*	276	*P2RX6*
2	*ANO10*	57	*CACNG6*	112	*GABRB2*	167	*GRIN3A*	222	*KCNJ3*	277	*P2RX7*
3	*ANO2*	58	*CACNG7*	113	*GABRB3*	168	*GRIN3B*	223	*KCNJ4*	278	*PKD2*
4	*ANO3*	59	*CACNG8*	114	*GABRD*	169	*HCN1*	224	*KCNJ5*	279	*PKD2L1*
5	*ANO4*	60	*CATSPER1*	115	*GABRE*	170	*HCN2*	225	*KCNJ6*	280	*PKD2L2*
6	*ANO5*	61	*CATSPER2*	116	*GABRG1*	171	*HCN3*	226	*KCNJ8*	281	*RYR1*
7	*ANO6*	62	*CATSPER3*	117	*GABRG2*	172	*HCN4*	227	*KCNJ9*	282	*RYR2*
8	*ANO7*	63	*CATSPER4*	118	*GABRG3*	173	*HTR3A*	228	*KCNK1*	283	*RYR3*
9	*ANO8*	64	*CATSPERB*	119	*GABRP*	174	*HTR3B*	229	*KCNK10*	284	*SCN10A*
10	*ANO9*	65	*CATSPERD*	120	*GABRQ*	175	*HTR3C*	230	*KCNK12*	285	*SCN11A*
11	*AQP1*	66	*CATSPERG*	121	*GABRR1*	176	*HTR3D*	231	*KCNK13*	286	*SCN1A*
12	*AQP10*	67	*CFTR*	122	*GABRR2*	177	*HTR3E*	232	*KCNK15*	287	*SCN1B*
13	*AQP11*	68	*CHRNA1*	123	*GABRR3*	178	*HVCN1*	233	*KCNK16*	288	*SCN2A*
14	*AQP12A*	69	*CHRNA10*	124	*GJA1*	179	*ITPR1*	234	*KCNK17*	289	*SCN2B*
15	*AQP12B*	70	*CHRNA2*	125	*GJA10*	180	*ITPR2*	235	*KCNK18*	290	*SCN3A*
16	*AQP2*	71	*CHRNA3*	126	*GJA3*	181	*ITPR3*	236	*KCNK2*	291	*SCN3B*
17	*AQP3*	72	*CHRNA4*	127	*GJA4*	182	*KCNA1*	237	*KCNK3*	292	*SCN4A*
18	*AQP4*	73	*CHRNA5*	128	*GJA5*	183	*KCNA10*	238	*KCNK4*	293	*SCN4B*
19	*AQP5*	74	*CHRNA6*	129	*GJA6P*	184	*KCNA2*	239	*KCNK5*	294	*SCN5A*
20	*AQP6*	75	*CHRNA7*	130	*GJA8*	185	*KCNA3*	240	*KCNK6*	295	*SCN8A*
21	*AQP7*	76	*CHRNA9*	131	*GJA9*	186	*KCNA4*	241	*KCNK7*	296	*SCN9A*
22	*AQP8*	77	*CHRNB1*	132	*GJB1*	187	*KCNA5*	242	*KCNK9*	297	*SCNN1A*
23	*AQP9*	78	*CHRNB2*	133	*GJB2*	188	*KCNA6*	243	*KCNMA1*	298	*SCNN1B*
24	*ASIC1*	79	*CHRNB3*	134	*GJB3*	189	*KCNA7*	244	*KCNN1*	299	*SCNN1D*
25	*ASIC2*	80	*CHRNB4*	135	*GJB4*	190	*KCNB1*	245	*KCNN2*	300	*SCNN1G*
26	*ASIC3*	81	*CHRND*	136	*GJB5*	191	*KCNB2*	246	*KCNN3*	301	*TPCN1*
27	*ASIC4*	82	*CHRNE*	137	*GJB6*	192	*KCNC1*	247	*KCNN4*	302	*TPCN2*
28	*ASIC5*	83	*CHRNG*	138	*GJB7*	193	*KCNC2*	248	*KCNQ1*	303	*TRPA1*
29	*BEST1*	84	*CLCN1*	139	*GJC1*	194	*KCNC3*	249	*KCNQ2*	304	*TRPC1*
30	*BEST2*	85	*CLCN2*	140	*GJC2*	195	*KCNC4*	250	*KCNQ3*	305	*TRPC2*
31	*BEST3*	86	*CLCN3*	141	*GJC3*	196	*KCND1*	251	*KCNQ4*	306	*TRPC3*
32	*BEST4*	87	*CLCN4*	142	*GJD2*	197	*KCND2*	252	*KCNQ5*	307	*TRPC4*
33	*BSND*	88	*CLCN5*	143	*GJD3*	198	*KCND3*	253	*KCNS1*	308	*TRPC5*
34	*CACNA1A*	89	*CLCN6*	144	*GJD4*	199	*KCNF1*	254	*KCNS2*	309	*TRPC6*
35	*CACNA1B*	90	*CLCN7*	145	*GJE1*	200	*KCNG1*	255	*KCNS3*	310	*TRPC7*
36	*CACNA1C*	91	*CLCNKA*	146	*GLRA1*	201	*KCNG2*	256	*KCNT1*	311	*TRPM1*
37	*CACNA1D*	92	*CLCNKB*	147	*GLRA2*	202	*KCNG3*	257	*KCNT2*	312	*TRPM2*
38	*CACNA1E*	93	*CLIC1*	148	*GLRA3*	203	*KCNG4*	258	*KCNU1*	313	*TRPM3*
39	*CACNA1F*	94	*CLIC2*	149	*GLRA4*	204	*KCNH1*	259	*KCNV1*	314	*TRPM4*
40	*CACNA1G*	95	*CLIC3*	150	*GLRB*	205	*KCNH2*	260	*KCNV2*	315	*TRPM5*
41	*CACNA1H*	96	*CLIC4*	151	*GRIA1*	206	*KCNH3*	261	*LRRC8A*	316	*TRPM6*
42	*CACNA1I*	97	*CLIC5*	152	*GRIA2*	207	*KCNH4*	262	*LRRC8B*	317	*TRPM7*
43	*CACNA1S*	98	*CLIC6*	153	*GRIA3*	208	*KCNH5*	263	*LRRC8C*	318	*TRPM8*
44	*CACNA2D1*	99	*CNGA1*	154	*GRIA4*	209	*KCNH6*	264	*LRRC8D*	319	*TRPV1*
45	*CACNA2D2*	100	*CNGA2*	155	*GRID1*	210	*KCNH7*	265	*LRRC8E*	320	*TRPV2*
46	*CACNA2D3*	101	*CNGA3*	156	*GRID2*	211	*KCNH8*	266	*MCOLN1*	321	*TRPV3*
47	*CACNA2D4*	102	*CNGA4*	157	*GRIK1*	212	*KCNJ1*	267	*MCOLN2*	322	*TRPV4*
48	*CACNB1*	103	*CNGB1*	158	*GRIK2*	213	*KCNJ10*	268	*MCOLN3*	323	*TRPV5*
49	*CACNB2*	104	*CNGB3*	159	*GRIK3*	214	*KCNJ11*	269	*MIP*	324	*TRPV6*
50	*CACNB3*	105	*GABRA1*	160	*GRIK4*	215	*KCNJ12*	270	*NALCN*	325	*VDAC1*
51	*CACNB4*	106	*GABRA2*	161	*GRIK5*	216	*KCNJ13*	271	*P2RX1*	326	*VDAC2*
52	*CACNG1*	107	*GABRA3*	162	*GRIN1*	217	*KCNJ14*	272	*P2RX2*	327	*VDAC3*
53	*CACNG2*	108	*GABRA4*	163	*GRIN2A*	218	*KCNJ15*	273	*P2RX3*	328	*ZACN*
54	*CACNG3*	109	*GABRA5*	164	*GRIN2B*	219	*KCNJ16*	274	*P2RX4*		
55	*CACNG4*	110	*GABRA6*	165	*GRIN2C*	220	*KCNJ18*	275	*P2RX5*		

**Table 2:** List of ion channels genes showing differential expression in nevi vs. melanoma (*p* < 0.05), sorted according to the AUC value. The analysis was carried out in the GEO GDS1375 dataset.

No.	Gene Name	*t* Test Nevi vs. Melanoma	Ratio Melanoma vs. Nevi		AUC	No.	Gene Name	*t* Test Nevi vs. Melanoma	Ratio Melanoma vs. Nevi		AUC
*1*	*ANO1*	8.7 × 10^−18^	0.231	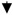	0.98	*47*	*CACNB2*	1.5 × 10^−3^	0.4388	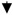	0.73
*2*	*KCNK7*	1.8 × 10^−17^	0.081	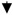	0.94	*48*	*TRPV6*	1.9 × 10^−3^	0.576	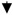	0.65
*3*	*SCNN1A*	1.0 × 10^−14^	0.07	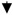	0.98	*49*	*CLIC2*	2.0 × 10^−3^	0.319	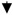	0.87
*4*	*SCNN1B*	1.4 × 10^−14^	0.313	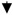	0.96	*50*	*SCN3A*	2.2 × 10^−3^	0.382	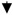	0.64
*5*	*GABRE*	2.3 × 10^−13^	0.203	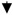	0.95	*51*	*GRIN2A*	2.3 × 10^−3^	0.619	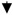	0.69
*6*	*GJB5*	3.4 × 10^−13^	0.1277	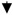	0.97	*52*	*CACNG4*	2.6 × 10^−3^	1.317	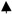	0.74
*7*	*CLIC1*	1.9 × 10^−12^	1.738	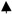	0.95	*53*	*GABRB1*	3.0 × 10^−3^	0.698	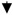	0.69
*8*	*KCND3*	2.0 × 10^−11^	0.487	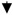	0.94	*54*	*GJB4*	3.7 × 10^−3^	0.588	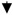	0.63
*9*	*KCNK1*	8.6 × 10^−11^	0.269	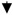	0.93	*55*	*CACNB4*	4.3 × 10^−3^	0.474	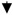	0.66
*10*	*GRIA1*	8.7 × 10^−11^	0.1049	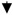	0.93	*56*	*CACNA11*	5.0 × 10^−3^	0.629	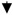	0.70
*11*	*VDAC1*	2.0 × 10^−10^	1.8	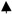	0.96	*57*	*KCNK2*	5.0 × 10^−3^	0.512	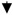	0.64
*12*	*GJB3*	2.1 × 10^−10^	0.2453	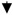	0.96	*58*	*TRPC1*	5.4 × 10^−3^	0.665	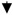	0.70
*13*	*KCNN2*	3.6 × 10^−10^	4.337	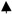	0.91	*59*	*TRPC7*	5.7 × 10^−3^	1.396	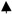	0.74
*14*	*AQP1*	5.5 × 10^−10^	0.268	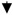	0.84	*60*	*GRIK1*	6.3 × 10^−3^	1.885	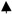	0.72
*15*	*ITPR3*	1.1 × 10^−9^	2.311	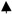	0.93	*61*	*GRIN2D*	6.7 × 10^−3^	1.922	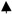	0.75
*16*	*RYR1*	1.8 × 10^−9^	0.2414	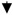	0.81	*62*	*CLIC4*	6.8 × 10^−3^	0.671	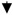	0.69
*17*	*GJA1*	2.1 × 10^−9^	0.086	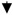	0.98	*63*	*CACNA1S*	7.7 × 10^−3^	1.91	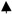	0.77
*18*	*KCNJ13*	1.9 × 10^−8^	0.266	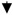	0.86	*64*	*CATSPER2*	8.1 × 10^−3^	0.677	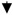	0.75
*19*	*TRPV2*	3.0 × 10^−8^	1.77	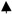	0.88	*65*	*GABRP*	8.1 × 10^−3^	0.3014	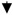	0.69
*20*	*GJB1*	3.4 × 10^−8^	2.643	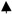	0.93	*66*	*GJA4*	9.0 × 10^−3^	0.5357	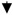	0.68
*21*	*CLIC3*	4.0 × 10^−8^	0.118	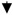	0.85	*67*	*KCNJ15*	9.0 × 10^−3^	0.506	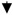	0.64
*22*	*LRRC8B*	4.4 × 10^−8^	0.459	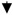	0.90	*68*	*CNGB3*	1.0 × 10^−2^	0.63	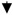	0.69
*23*	*HCN2*	2.1 × 10^−7^	3.88	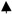	0.89	*69*	*SCNN1D*	1.0 × 10^−2^	0.735	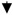	0.69
*24*	*TRPM1*	3.0 × 10^−7^	0.368	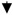	0.77	*70*	*GRIA4*	1.2 × 10^−2^	0.71	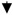	0.68
*25*	*BEST2*	3.6 × 10^−7^	0.456	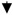	0.86	*71*	*TRPM3*	1.3 × 10^−2^	0.762	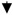	0.72
*26*	*AQP3*	6.9 × 10^−7^	0.067	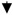	0.97	*72*	*CHRNA10*	1.5 × 10^−2^	0.759	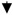	0.68
*27*	*KCNN4*	1.5 × 10^−6^	3.79	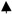	0.93	*73*	*AQP9*	1.9 × 10^−2^	0.56	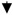	0.75
*28*	*SCN1B*	1.5 × 10^−6^	0.657	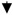	0.83	*74*	*VDAC3*	1.9 × 10^−2^	1.27	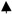	0.78
*29*	*LRRC8E*	1.5 × 10^−6^	0.563	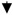	0.82	*75*	*KCNV1*	2.0 × 10^−2^	1.737	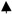	0.70
*30*	*CACNB1*	2.9 × 10^−6^	0.614	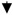	0.82	*76*	*VDAC2*	2.0 × 10^−2^	1.15	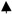	0.71
*31*	*KCNJ12*	3.5 × 10^−6^	0.388	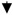	0.80	*77*	*CHRNB1*	2.1 × 10^−2^	0.754	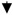	0.68
*32*	*KCNJ18*	3.5 × 10^−6^	0.388	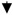	0.80	*78*	*ANO2*	2.4 × 10^−2^	0.67	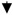	0.68
*33*	*TRPM4*	3.6 × 10^−6^	0.154	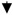	0.87	*79*	*CHRNB2*	2.6 × 10^−2^	1.383	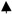	0.70
*34*	*KCNS3*	7.7 × 10^−6^	0.403	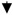	0.93	*80*	*SCNN1G*	2.8 × 10^−2^	1.45	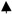	0.67
*35*	*PKD2L1*	8.7 × 10^−6^	2.66	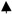	0.86	*81*	*TRPC6*	2.8 × 10^−2^	0.594	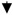	0.67
*36*	*P2RX4*	1.0 × 10^−5^	1.756	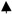	0.85	*82*	*KCNA1*	3.0 × 10^−2^	0.79	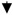	0.67
*37*	*GRIK2*	3.9 × 10^−5^	3.922	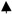	0.86	*83*	*PKD2L2*	3.0 × 10^−2^	0.447	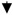	0.52
*38*	*KCNJ4*	5.0 × 10^−5^	2.87	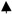	0.84	*84*	*GABRA6*	3.9 × 10^−2^	0.622	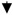	0.65
*39*	*TRPM2*	6.5 × 10^−5^	0.219	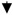	0.93	*85*	*GRINB2*	3.9 × 10^−2^	1.225	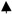	0.67
*40*	*CLCN7*	1.2 × 10^−4^	1.753	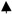	0.80	*86*	*KCNJ2*	4.0 × 10^−2^	1.33	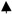	0.72
*41*	*MCOLN3*	2.2 × 10^−4^	0.352	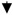	0.62	*87*	*TRPV5*	4.0 × 10^−2^	1.29	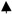	0.66
*42*	*SCN2B*	2.6 × 10^−4^	0.483	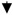	0.71	*88*	*ASIC1*	4.1 × 10^−2^	0.71	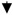	0.63
*43*	*LRRC8D*	2.8 × 10^−4^	1.39	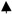	0.78	*89*	*AQP5*	4.1 × 10^−2^	1.498	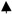	0.67
*44*	*MCOLN1*	3.8 × 10^−4^	1.43	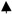	0.72	*90*	*CACNG3*	4.3 × 10^−2^	1.903	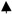	0.73
*45*	*CLCN6*	5.0 × 10^−4^	0.74	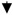	0.79	*91*	*TRPC4*	4.3 × 10^−2^	2.097	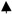	0.69
*46*	*CNGA1*	5.0 × 10^−4^	0.58	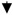	0.72						

**Table 3:** Ion channels genes showing very high discriminating ability (AUC > 0.90) in the Talantov dataset were validated in a first round validation in the Raskin dataset. Genes passing the first validation were then validated in the Riker dataset. Genes passing screening phase and all two validations were searched in Pubmed to identify those never directly related to melanoma. Genes showing 0 value in the “Novelty” column are genes never related to melanoma according to Pubmed abstract. Five genes were selected according to this procedure (*SCNN1A*, *GJB3*, *KCNK7*, *GJB1*, *KCNN2*). Empty cells indicate lack of validation.

No.	Gene Name	Screening Phase (in the Talantov Dataset, GEO, GDS1375)			First-Round Validation (in the Raskin Dataset, GEO GSE15605) *	Second-Round Validation (in the Riker Dataset, Oncomine) **	Novelty (in PubMeds Abstracts) ***	Full in Silico Validation
		63 Patients			62 Patients	59 Patients		
		*t* Test Melanoma vs. Nevi	Ratio Melan. vs. Nevi	AUC	Validation on AUC Value *	Validation on Ratio Value	Gene Name and Melanoma Words Co-Occurrence	
1	*SCNN1A*	1.0 × 10^−14^	0.07	0.98	Yes (0.85)	Yes (−4.94)	0	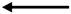
2	*ANO1*	8.6 × 10^−18^	0.231	0.98	Yes (0.87)			
3	*GJA1*	2.1 × 10^−9^	0.086	0.98	Yes (0.88)			
4	*GJB5*	3.4 × 10^−13^	0.1277	0.97				
5	*GJB3*	2.1 × 10^−10^	0.2453	0.96	Yes (0.86)	Yes (−6.662)	0	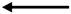
6	*AQP3*	6.9 × 10^−7^	0.067	0.97				
7	*SCNN1B*	1.4 × 10^−14^	0.313	0.96	Yes (0.87)			
8	*VDAC1*	2.0 × 10^−10^	1.8	0.96				
9	*CLIC1*	1.9 × 10^−12^	1.738	0.96				
10	*GABRE*	2.3 × 10^−13^	0.203	0.95	Yes (0.88)	Yes (−3.162)	≥1	
11	*KCNK7*	1.8 × 10^−17^	0.081	0.94	Yes (0.86)	Yes (−2.832)	0	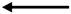
12	*KCND3*	2.0 × 10^−11^	0.487	0.94	Yes (0.89)			
14	*KCNN4*	1.5 × 10^−6^	3.79	0.93				
13	*ITPR3*	1.1 × 10^−9^	2.311	0.93				
15	*KCNK1*	8.6 × 10^−11^	0.269	0.93	Yes (0.89)			
16	*KCNS3*	7.7 × 10^−6^	0.403	0.93				
17	*TRPM2*	6.5 × 10^−5^	0.219	0.93				
18	*GRIA1*	8.7 × 10^−11^	0.1049	0.93				
19	*GJB1*	3.4 × 10^−8^	2.643	0.93	Yes (0.87)	Yes (3.303)	0	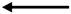
20	*KCNN2*	3.6 × 10^−10^	4.337	0.91	Yes (0.91)	Yes (2.284)	0	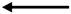

* validation in GSE15605 was considered effective if AUC > 0.85 (AUC is reported in parenthesis); ** validation in Riker dataset was considered effective if difference of melanoma vs. normal skin is <0.5 or >1.5 (difference is reported in parenthesis, calculated from log_2_ median-centered intensity according to Oncomine); *** Novelty was assessed onto the five genes validated in the first and second validations steps.

**Table 4:** Gene-drug interaction according to the Drug Gene Interaction Database (www.dgidb.org). The five genes selected in Table 3 are investigated. Drugs known to target the given genes and interaction type are according to www.dgidb.org. Main target tissues, pharmacological action and IUPAC (International Union of Pure and Applied Chemistry) are derived from Drugbank (https://www.drugbank.ca/drugs). EC50 of the indicated pharmacological actions are also reported with corresponding reference. References indicating the ion channel/drug interaction are reported.

Gene Name	Drug Targeting the Gene, According to www.dgidb.org, and IUPAC Name	Reference (PMID)	Interaction Type with the Gene	Main Target Tissue	Action and Indications	EC50
*SCNN1A*	Triamterene 6-phenylpteridine-2,4,7-triamine	[30]	channel blocker	kidney	diuretic, anti-edema	1660 nM [31]
	Amiloride 3,5-diamino-6-chloro-N-(diaminomethylidene)pyrazine-2-carboxamide	[32]	channel blocker	kidney	diuretic; congestive heart failure; hypertension.	0.1 μM [33]
*GJB3*	Flufenamic acid 2-{[3-(trifluoromethyl)phenyl]amino}benzoic acid	[34]	inhibitor	muscles joints	anti-cancer	100/200 μM [35]
	Carbenoxolone 2S,4aS,6aR,6aS,6bR,8aR,10S,12aS,14bR)-10-(3-carboxypropanoyloxy)-2,4a,6a,6b,9,9,12a-heptamethyl-13-oxo-3,4,5,6,6a,7,8,8a,10,11,12,14b-dodecahydro-1H-picene-2-carboxylic acid	[34,36]	inhibitor	digestive tract	anti-ulcer/neuro protection	48 μM [37]
*KCNK7*	Not found					
*GJB1*	Not found					
*KCNN2*	Miconazole 1-[2-(2,4-dichlorophenyl)-2-[(2,4-dichlorophenyl)methoxy]ethyl]-1H-imidazole	[38]	Inhibitor	systemic, skin	anti-fungal infections	75 μM [39]
	Tubocurarine (1S,16R)-9,21-dihydroxy-10,25-dimethoxy-15,15,30-trimethyl-7,23-dioxa-15,30-diazaheptacyclo [22.6.2.2^3^,^6^.1^8^,^12^.1^18^,^22^.0^27^,^31^.0^16^,^34^]hexatriaconta-3,5,8(34),9,11,18(33),19,21, 24, 26,31,35-dodecaen-15-ium	[34]	channel blocker	CNS	diagnostic in myastenia gravis; to treat smoking withdrawl syndrom	1.3 μM [40]
	Bendroflumethiazide 3-benzyl-1,1-dioxo-6-(trifluoromethyl)-3,4-dihydro-2H-1λ^6^,2,4-benzothiadiazine-7-sulfonamide	[34]	channel blocker	kidney smooth muscle cells	Anti HBV, diuretic; anti-edema; hypertension	53 μM [41]

**Table 5:** Top 10 biological processes enriched in a statistically significant way by investigating in a STRING “multiple proteins” search the following molecules: CYP51A1, NOS3, NOS2, NOS1, KCNMA1, KCNMB3, KCNN4, KCNH2, KCNH7, KCNH6, KCNN2.

Biological Process	Pathway ID	False Discovery Rate
Potassium ion transmembrane transport	GO:00781805	1.9 × 10^−7^
Arginine catabolic process	GO:0006527	6.4 × 10^−6^
Synaptic transmission	GO:0007268	6.4 × 10^−6^
Positive regulation of guanylate cyclase activity	GO:0031284	4.2 × 10^−5^
Regulation of system process	CO:0044057	4.2 × 10^−5^
Nitric oxide biosynthetic process	GO:0006809	5.3 × 10^−5^
Regulation of potassium ion transport	GO:0043266	5.7 × 10^−5^
Regulation of blood circulation	GO:1903522	5.7 × 10^−5^
Nitric oxide mediated signal transduction	GO:0007263	8.1 × 10^−5^
Blood circulation	GO:0008015	8.3 × 10^−5^
